# Lignin Metabolism Is Crucial in the Plant Responses to *Tambocerus elongatus* (Shen) in *Camellia sinensis* L.

**DOI:** 10.3390/plants14020260

**Published:** 2025-01-17

**Authors:** Wenli Wang, Xiaogui Zhou, Qiang Hu, Qiuhong Wang, Yanjun Zhou, Jingbo Yu, Shibei Ge, Lan Zhang, Huawei Guo, Meijun Tang, Xin Li

**Affiliations:** Key Laboratory of Tea Quality and Safety Control, Ministry of Agriculture and Rural Affairs, Tea Research Institute, Chinese Academy of Agricultural Sciences, Hangzhou 310008, Chinazxg@tricaas.com (X.Z.); lixin@tricaas.com (X.L.)

**Keywords:** *Camellia sinensis*, flavonoids, insect pest, jasmonic acid, lignin, phenylpropanoid pathway, plant defense

## Abstract

*Tambocerus elongatus* (Shen) (Hemiptera: Cicadellidae) is a devastating insect pest species of *Camellia sinensis*, significantly affecting the yield and quality of tea. Due to growing concerns over the irrational use of insecticides and associated food safety, it is crucial to better understand the innate resistance mechanism of tea trees to *T. elongatus*. This study aims to explore the responses of tea trees to different levels of *T. elongatus* infestation. We first focused on the primary metabolism and found that the amino acid levels decreased significantly with increasing *T. elongatus* infestation, while sugar accumulation showed an opposite trend. Moreover, secondary metabolite analysis showed a significant increase in flavonoid compounds and lignin content after *T. elongatus* infestation. Metabolomics analysis of the flavonoid compounds revealed a decrease in the proanthocyanidin level and an increase in anthocyanidin glycosides (anthocyanins and their derivatives) after *T. elongatus* infestation. *T. elongatus* infestation also caused a decrease in the abundance of non-ester catechins and an increase in the abundance of ester catechins. Furthermore, the gene expression analysis revealed that transcripts of genes involved in flavonoid biosynthesis, such as *CsCHI*, *CsF3H*, *CsF3′H*, *CsFNS*, *CsFLS*, and *CsUFGT*, were down-regulated, while genes involved in the lignin pathway were up-regulated by insect infestation, suggesting that lignin probably plays a pivotal role in tea plant response to *T. elongatus* infestation. Analysis of the expression of related genes indicates that the jasmonate (JA) pathway primarily responds to leafhopper damage. These findings suggest that the lignin pathway and JA play a preferential role in tea plant response to *T. elongatus*. Furthermore, the production of saccharides and the accumulation of anthocyanin glycosides in the flavonoid metabolic pathway are critical during this stress response. Further exploration of the roles of anthocyanin glycosides and lignin in tea tree resistance could provide a theoretical basis for understanding the defense mechanism of tea trees against *T. elongatus* damage.

## 1. Introduction

Tea [*Camellia sinensis* (L.) O. Kuntze] is a perennial woody cash crop, harvested mainly for its leaf buds and young leaves, and is known as one of the world’s three most important non-alcoholic beverages, together with coffee and cocoa [1]. Tea plants are widely grown in the warm and humid regions of the tropics and subtropics [2]. It is favored by people from all classes beyond geographical location, ethnicity, and identity because of its unique flavor, taste, and rich medicinal value attributed to tea polyphenols, amino acids, caffeine, and other substances beneficial to human health [3,4].

New shoots, especially the young leaves and apical leaf buds of the tea tree, are used as raw materials for processing tea. The quality of tea is closely related to the contents of various metabolites, including some specialized metabolites accumulated in fresh leaves of the tea tree. However, many pests (such as tea green leafhopper and tea geometrid) prefer feeding on young and tender tea leaves, severely affecting tea quality and yield [5]. Although pesticides are the primary means of pest control, they can potentially lead to issues related to the quality and safety of tea. As China has strengthened its focus on tea quality and food safety, the deployment of the tea tree’s inherent resistance to control pest infestation and stimulate plant intrinsic defense mechanisms that elicit a spectrum of defense responses is pivotal to integrated pest management in tea plantations [6,7]. Notably, the resistance of tea trees to pests and diseases is largely dependent on defense compounds such as natural polyphenols and plant hormones such as jasmonic acid (JA) and salicylic acid (SA) [8].

Lignin is a major secondary cell wall component that provides mechanical support to plants and is also a chemical defense compound against biotic stress [9,10]. When a plant is infested with pests, tissue-specific lignin production and accumulation occur at specific locations to enhance the mechanical strength of the plant cell wall, thus forming a physical barrier. Such a barrier effectively mitigates a variety of external stresses and gives the plant the ability to defend itself against pests and pathogens [10,11]. Studies have shown that the enhanced lignin biosynthesis in plants contributes to their resistance against citrus canker and aphid infestation [12,13]. A relatively high accumulation of lignin increases the strength of birch leaves, which reduces vulnerability to the autumnal moth [14]. The synthesis and accumulation of SA and lignin facilitate resistance against the brown planthopper in rice [15]. SA and JA are signaling compounds in plants that play critical roles in defense mechanisms against microbial pathogens and insect herbivores [16,17]. Numerous studies have demonstrated SA induces greater defense against stinging insects, and JA is mainly involved in defense against leaf-chewing herbivores [18,19,20]. Recent studies have revealed that JA is also involved in the lignification process of plant cells. For example, exogenous methyl jasmonate (MeJA) treatment enhances lignin deposition and cell wall damage thickening in Arabidopsis and mechanical injury to plants causes a rapid increase in JA content and lignin synthesis [21,22]. SA also induces the up-regulation of genes associated with lignin synthesis. This up-regulation leads to the enhanced production of lignin, which is key for plant disease resistance. Previous research has shown that SA can up-regulate the expression of *CsCAD1* and *CsCAD2* in tea leaves, and *CsCAD1* plays a key role in lignin biosynthesis [8,23,24,25].

Leafhoppers represent a significant category of crop pests, primarily inflicting damage by feeding on the sap of plant leaves and causing mechanical harm through egg-laying activities [26]. Moreover, certain leafhopper species are capable of transmitting plant viruses, exacerbating their threat to agricultural productivity [27]. In China, the tea industry is particularly susceptible to damage from several leafhopper species, with the most destructive being *Empoasca onukii* (Matsuda) (Hemiptera: Cicadellidae), *Chanohirata theae* (Matsumura) (Hemiptera: Cicadellidae), and *Tambocerus elongatus* (Shen) (Hemiptera: Cicadellidae) [28,29]. According to our observation, the *T. elongatus* is approximately twice the length of the *Empoasca onukii*, and it prefers to congregate at the bud tips for feeding, where it consumes a substantial amount of the tea bud’s sap. Nevertheless, the damage symptoms inflicted by *T. elongatus* are relatively milder compared to those inflicted by the *Empoasca onukii*.

To date, a considerable amount of research has been conducted on *Empoasca onukii* infesting tea trees, whereas *T. elongatus* has received little attention. In this study, we investigated the changes in key primary and secondary metabolites in tea leaves in response to *T. elongatus* infestation in tea leaves, focusing on the phenylpropanoid metabolic pathway in tea trees. In addition, we unveiled the role of defense hormones under varying levels of pest infestation by examining the expression levels of JA- and SA-related genes to further probe into the mechanism of tea plants’ resistance to *T. elongatus*, thereby providing a theoretical basis for the green management of tea plantation pests.

## 2. Results

### 2.1. Changes in Primary Metabolites in Tea Leaves Under Varying Levels of T. elongatus Infestation

**Figure 1:**
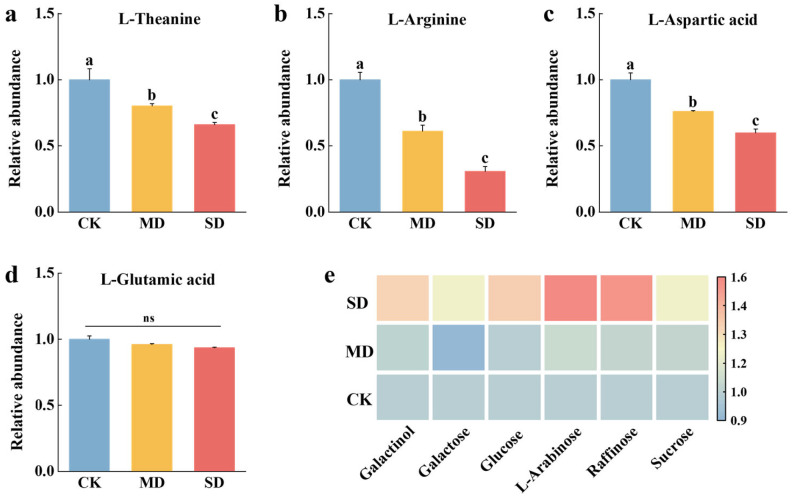
*T. elongatus* feeding affects the abundance of major amino acids and sugars in tea leaves. Relative abundance of (**a**). Theanine; (**b**). Arginine; (**c**). Aspartic acid; (**d**). Glutamic acid; and (**e**). Major sugars in tea buds. The color gradient from red to blue indicates the abundance of sugars from high to low. CK: control, no larval infestation; MD: moderate infestation level, 5 larvae per bud; SD: severe infestation level, 10 larvae per bud. The relative abundance of various amino acids was determined based on the peak areas from the metabolomics results. The mean denoted by the different lower-case letters indicates statistically significant differences between the treatments according to Duncan’s Multiple Range Test (DMRT) at *p* < 0.05, where ns represents non-significant and error bars indicate standard deviation.

Feeding by *T. elongatus* significantly reduced the accumulation of theanine, arginine, and aspartic acid in fresh tea leaves (Figure 1). The abundance of L-theanine decreased by 20% and 34% in MD and SD, respectively (Figure 1a). Similarly, the arginine level decreased by 39% in MD and a more substantial decrease of 70% in SD (Figure 1b), while the aspartic acid decreased by 24% in MD and 41% in SD (Figure 1c). Although the abundance of glutamic acid also decreased, however, this reduction was not statistically significant (Figure 1d). In addition, saccharides showed a significant increase following T. elongatus feeding (Figure 1e).

### 2.2. Changes in Secondary Metabolites in Tea Leaves Under Varying Levels of T. elongatus Infestation

**Figure 2:**
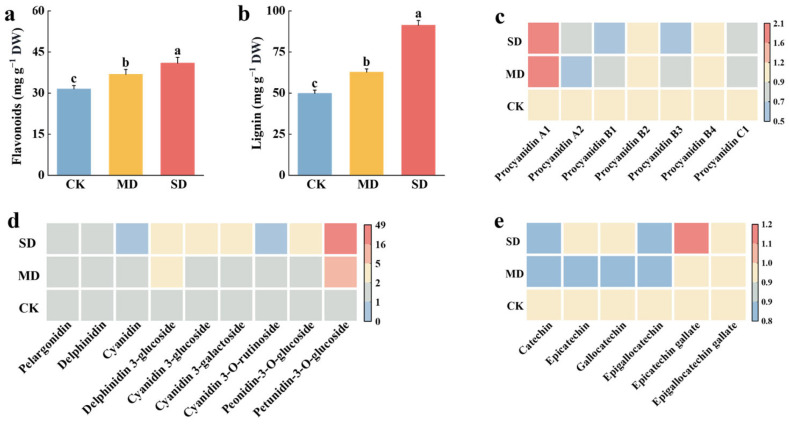
Flavonoid, total lignin, and flavonoid-targeted metabolite contents in different infestation levels of *T. elongatus*. (**a**). Content of total flavonoids. (**b**). Content of total lignin. (**c**). Abundance of Proanthocyanidin. (**d**). Abundance of anthocyanins and their glycosides. (**e**). Abundance of the main catechins. CK: control, no larval infestation; MD: moderate infestation level, 5 larvae per bud; SD: severe infestation level, 10 larvae per bud. The color gradient from red to blue indicates the abundance from high to low. The data denoted by the different lower-case letters indicated significant differences between the treatments (DMRT, *p* < 0.05) and error bars indicate standard deviation. The content values are based on the dry weight of tea leaves.

Upon exposure to *T. elongatus* infestation, tea plants exhibit a marked increase in hydrogen peroxide (H_2_O_2_) content and malondialdehyde (MDA) content (Figure S1), which indicates that the tea plant has incurred damage, while the levels of secondary metabolites, flavonoids, and lignin were significantly higher in tea buds with increasing *T. elongatus* infestation (Figure 2a,b). Specifically, compared with the control group, leaf flavonoids increased by 16.8% in the moderate infestation level (MD, 5 larvae per shoot) and 29.9% in the severe infestation level (SD, 10 larvae per shoot), while the leaf lignin content increased by 26.0% in MD and 83.3% in SD, revealing that infestation by the *T. elongatus* led to an increase in both the flavonoid and lignin contents in tea leaves. Nonetheless, the increase in the lignin content was greater than the increase in flavonoids. The metabolome data revealed a decreasing trend in the proanthocyanidin content with increasing leafhopper infestation. However, the proanthocyanidin A1 content increased (Figure 2c). Additionally, the anthocyanidin content showed a decreasing trend among the anthocyanins and their derivatives, while their glycoside derivatives showed an increasing trend (Figure 2d). Among the catechin substances, only epicatechin gallate (ECG) and epigallocatechin gallate (EGCG) showed an increasing trend, while catechin (C) and epigallocatechin (EGC) showed a decreasing trend. Epicatechin (EC) and gallocatechin (GC) showed a decreasing trend followed by an increasing trend with increasing *T. elongatus* infestation. This implies that the content of non-ester catechins decreased, and the content of ester catechins increased under *T. elongatus* infestation (Figure 2e).

### 2.3. Gene Expression in the Phenylpropanoid Pathway Changed with Varying Levels of T. elongatus Infestation

**Figure 3:**
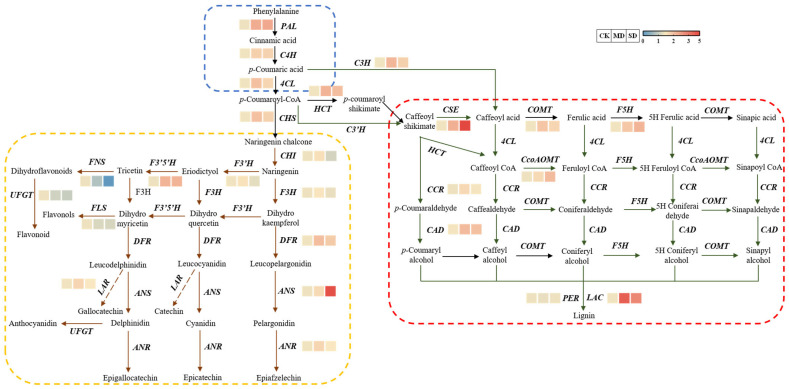
Expression of genes involved in the phenylpropanoid metabolic pathway. PAL, Phenylalanine ammonia-lyase; C4H, Cinnamic acid 4-hydroxylase; 4CL, 4-Coumarinyl-CoA ligase; CHS, Chalcone synthase; CHI, Chalcone isomerase; F3H, Flavanone 3-hydroxylase; F3′H, Flavonoid 3′-hydroxylase; F3′5′H, Flavonoid 3′5′-hydroxylase; FNS, Flavonoid synthase; FLS, Flavonol synthetase; UFGT, UDP-glycose flavonoid glycosyltransferase; DFR, Dihydroflavonol reductase; ANS, Anthocyanin synthase; ANR, Afsnthocyanidin reductase; LAR, Leucoanthocyanidin reductase; HCT, Hydroxycinnamoyl acyltransferase; C3H, p-Coumaric acid 3 hydroxylase; CSE, Caffeoyl shikimate esterase; COMT, Caffeic acid O-methyltransferase; F5H, Ferulic acid 5-hydroxylase; CcoAOMT, Caffeoyl-CoA-O-methyltransferase; CCR, Coumarin-CoA reductase; CAD, Cinnamyl alcohol dehydrogenase; PER, Peroxidase; LAC, Laccase. CK: control, no larval infestation; MD: moderate infestation level, 5 larvae per bud; SD: severe infestation level, 10 larvae per bud. The color gradient from red to blue indicates the relative transcript expression levels from high to low.

After leafhopper infestation, the transcript levels of downstream genes in the flavonoid pathway such as *CsDFR*, *CsANS*, *CsANR*, *CsF3′5′H*, and *CsLAR* were up-regulated, while *CsCHI*, *CsF3H*, *CsF3′H*, *CsFNS*, *CsFLS*, and *CsUFGT* were down-regulated. Similarly, the key genes in the lignin synthesis pathway such as *CsC3H*, *CsCSE*, *CsCOMT*, *CsF5H*, *CsCCR*, *CsCAD*, *CsCcoAOMT*, and *CsLAC* were up-regulated (Figure 3). This phenomenon is consistent with the higher increase in the lignin content than flavonoids in Figure 2.

### 2.4. Expression of Key Genes Related to Insect Resistance Hormones

**Figure 4:**
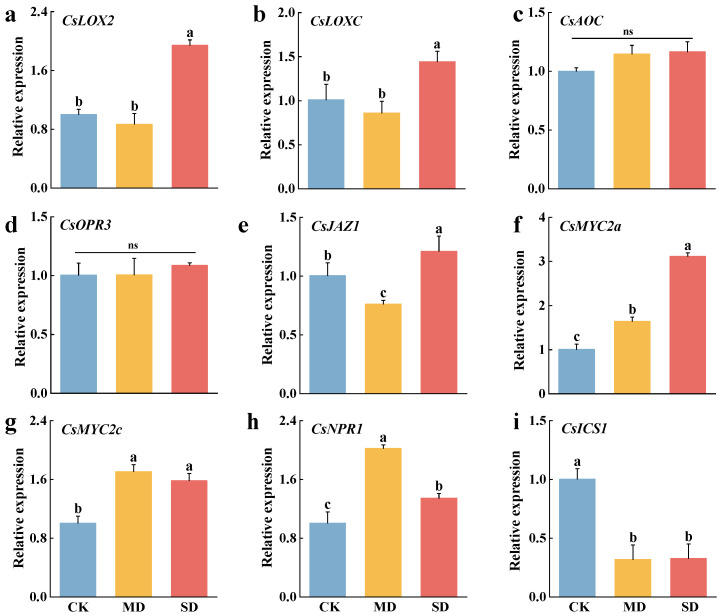
Hormone-related gene expression. (**a**). LOX2, lipoxygenase 2; (**b**). LOXC, lipoxygenase C; (**c**). AOC, allene oxide cyclase; (**d**). OPR3, 12-oxophytodienoate reductase; (**e**). JAZ1, JAZ protein 1, a key transcriptional repressor during JA signaling; (**f**). MYC2a, the JA pathway MYC2a transcription factors; (**g**). MYC2c, the JA pathway MYC2c transcription factors; (**h**). NPR1, Nonexpressor of pathogenesis-related genes 1 in SA pathway; (**i**). ICS1, isochorismate synthase 1 in SA pathway. CK: control, no larval infestation; MD: moderate infestation level, 5 larvae per bud; SD: severe infestation level, 10 larvae per bud. The data denoted by the different lower-case letters indicated significant differences between the treatments (DMRT, *p* < 0.05), where ns represents non-significant and error bars indicate standard deviation.

The results showed a significant increase in the expression of both lipoxygenase 2 (*CsLOX2*) and lipoxygenase C (*CsLOXC*) in the JA pathway in SD (Figure 4). The expression of allene oxide cyclase (*CsAOC*) and 12-oxophytodienoate reductase (*CsOPR3*) showed an increasing trend, although not statistically significant. However, the expression of the negative regulator JAZ protein 1 (JAZ1) significantly decreased in MD but increased in SD. Additionally, MYC2a transcription factors (*CsMYC2a*) and MYC2c transcription factors (*CsMYC2c*) were significantly increased in SD, while *CsMYC2a* was significantly increased in SD. The expression of *CsMYC2a* and *CsMYC2c* transcription factors was significantly increased in both MD and SD. Nonexpressor of pathogenesis-related genes 1 (*CsNPR1*) is a known master regulator of SA-mediated defense responses. The expression of *CsNPR1* increased significantly after *T. elongatus* infestation, showing a tendency to increase and then decrease. However, the expression of *CsNPR1* after *T. elongatus* infestation was significantly higher than that of CK in all cases. In the other pathway of SA synthesis, the expression of isochorismate synthase 1 (*CsICS1*), encoding a key enzyme in the pathway, significantly decreased in both MD and SD.

## 3. Discussion

The leafhopper is a major herbivore that infests tea trees, leading to considerable losses in both the quantity and quality of tea harvests on an annual basis [30]. The present study aimed to examine the response of tea trees to different levels of infestation by a recently revealed leafhopper, *T. elongatus*. Therefore, the changes in primary and secondary metabolites, as well as the transcript levels of genes related to defense hormones, were analyzed in tea leaves after different levels of infestation with *T. elongatus* on the tea cultivar ‘Longjing 43’.

### 3.1. T. elongatus Infestation Suppresses Primary Metabolism but Enhances Antioxidant Defense

Plants under biotic stress experience a considerable surge in H_2_O_2_ and MDA levels, concurrently developing defensive mechanisms at the cost of other essential functions, such as growth and reproduction [31]. Reactive oxygen species (ROS) exhibit a dual role based on spatiotemporal dynamics: at high concentrations, they act as an inducer of oxidative stress, while transient, modest increases serve crucial signaling functions [32]. To mitigate harmful ROS levels under stress, plants activate their antioxidant defense systems, which include non-enzymatic antioxidants derived from the secondary metabolism. Likewise, when plants are attacked by insects or pathogens, they usually increase their secondary metabolism to protect themselves against invaders. At the same time, the primary metabolism is often suppressed [33]. In this study, we found that infestation by *T. elongatus* led to a decrease in the content of major amino acids, which are the primary metabolites of tea. These findings align with the previous study on the little green leafhopper, which demonstrated a decrease in theanine, glutamic acid, aspartic acid, and serine, among other major amino acids, in tea following an attack or mechanical damage by the little green leafhopper [31]. In addition, studies have demonstrated that sugars serve not only as a substrate for starch synthesis but also as a signal transducer [34]. For instance, sucrose has been found to induce senescence in isolated tobacco leaves [35]. Our data on metabolite analysis showed an increase in the saccharide content, which may be attributed to their role in inducing signaling related to insect resistance.

### 3.2. T. elongatus Infestation Preferentially Up-regulates the Expression of Genes Involved in Lignin Biosynthesis over Flavonoid Biosynthesis

The phenylpropanoid metabolic branch of the secondary metabolic pathway is associated with biotic stress and it consists of two main pathways: the lignin pathway and the flavonoid pathway [36]. HCT and CHS are crucial enzymes located at critical branch points of the lignin and flavonoid pathways. Their gene expression regulates the flow of carbon sources in these pathways and affects plant responses to external stresses [3]. Meanwhile, under stress, carbon source homeostasis is disrupted. This metabolic flow can result in the up-regulation of the lignin pathway. We found that the expression of *CsCHS* and *CsHCT* increased in MD and SD in response to *T. elongatus* in tea. These results suggest that lignins are more responsive than flavonoids to such insect infestation. Therefore, it is likely that lignin plays a more crucial role in the response of tea trees to infestation by *T. elongatus*. In addition, research has indicated that when the *Empoasca onukii* feeds on ‘Huangjinya’ and ‘Fud-ingdabaicha’ varieties, it triggers an up-regulation of proteins associated with lignin biosynthesis. Additionally, the expression patterns of lignin synthesis-related genes (*CsLAC1*, *CsLAC2*, *CsCCR1*, *CsCAD1*, *CsCAD3*, and *CsCAD4*) manifest as an initial increase in expression levels, followed by a sharp decline, and subsequently another upsurge [37]. It indicates a complex pattern of gene regulation in response to leafhopper infestation. Kaempferol glucoside contributes to the response of tea trees to cold stress [38]. Moreover, studies have shown that *CsUGT89AC1* through the JA pathway glycosylates quercetin glucosides in response to *E. grisescens* infestation, and quercetin glucosides enhance tea tree defense against herbivores [5]. This aligns with the findings in our study, where a notable increase in anthocyanin glycoside compounds was observed following *T. elongatus* infestation, suggesting that anthocyanin glycoside substances contribute to the plant’s defense against insect pests.

### 3.3. Tea Plants Respond to T. elongatus Infestation by Activating SA and JA Signaling Pathways

Phytohormones play a crucial role in plant resistance to biotic stress [16]. JA, a lipid-derived signaling molecule, can be activated when plants are attacked by insects. JA induces the synthesis of insect-resistant compounds and the formation of insect-resistant tissues in plants, which adversely affects feeding, physiological activities, and host selection of pests, thereby producing a direct insect-resistant defense response [39,40,41]. Our study suggests that *T. elongatus* infesting tea leaves activates JA signaling to confer defense to tea plants. It has been shown that the mechanical wounding caused by tea geometrids can induce the synthesis of JA [42,43], which is consistent with the results of this paper. The JA pathway also actively enhances the tea plant’s defense against tea geometrids by positively regulating the activity of polyphenol oxidase [44]. In addition, the application of JA to cucumber resulted in leaf thickening, and a marked increase in trichome density and phenol levels, which bolstered resistance against *Liriomyza sativae*, and notably reduced the extent of damage caused by the pest [45]. In this experiment, the expression of *CsNPR1*, which is related to SA signaling, significantly increased after *T. elongatus* damage. However, the key gene of the isobranchial acid synthesis pathway, *CsICS1*, decreased significantly. This suggests that the SA signaling pathway is activated after leafhopper infestation, which may regulate the expression of SA-responsive genes, thereby playing a role in plant defense responses.

This study reveals that both lignins and flavonoids in the phenylpropanoid pathway are synthesized when tea trees are infested by *T. elongatus*. While lignins may act preferentially in defense, both compounds play a role and should be considered for further understanding the insect resistance mechanism in tea plants. Plant hormones frequently act interdependently through complex antagonistic or synergistic interactions [46,47]. At the hormonal level, in this investigation, it was revealed that JA assumes a more significant role than SA in response to *T. elongatus* infestation. Although it is generally believed that they have antagonistic roles, due to the complex regulatory network between hormones, JA and SA can also work together with other hormones through some transcription factors. This study reveals critical metabolic responses of tea plants to *T. elongatus* infestation and provides a theoretical basis for further utilizing the tea plant’s resistance mechanism to control *T. elongatus*, thereby inspiring new ideas for future studies.

## 4. Materials and Methods

### 4.1. Plant Materials and Treatments

The field study was conducted in July 2023 at the Tea Research Institute, Chinese Academy of Agricultural Sciences, Hangzhou, China. In this study, pest-free branches measuring 20 cm in length from the ‘Longjing 43’ tea cultivar were covered with miniature insect cages. Ten days post-enclosure, larvae of *T. elongatus*, those at the 3–4 instar stage, were directly captured from the field and randomly introduced into these cages. The experiment was set up with three distinct levels of infestation accounting for different numbers of insects: a control with no larval infestation (CK), a moderate infestation level (MD, 5 larvae per bud), and a severe infestation level (SD, 10 larvae per bud). Five days later, the samples were collected. Sampling was conducted in accordance with the criterion of one bud, one leaf. Bud tips exhibiting a moderate infestation level were selected from those displaying no symptom of reddening of the veins, while bud tips with a severe infestation level were selected from those exhibiting discoloration of the main veins, whole buds, or stems. Subsequently, approximately 15 bud tips (about 1–2 g) were collected with a single replicate, wrapped individually in aluminum foil, and promptly stored in liquid nitrogen to serve as samples for further analysis.

### 4.2. Determination of Total Flavonoid Content

The total flavonoid in tea leaves was extracted and measured following a previously described method [48]. The tea leaves samples were ground using liquid nitrogen in a mortar and pestle. Then, 0.2 g of the frozen sample was weighed and 3 mL methanol with 1% HCl and a little quartz sand were added to grind the sample into a homogenate under an ice bath. The homogenate was centrifuged at 4 °C for 20 min at 2000× *g*. The resulting supernatant was used to determine the flavonoid content. The reaction system for the determination of the flavonoid content consisted of 300 μL of 5% NaNO_2_, 300 μL of 10% AlCl_3_, and 300 μL of the supernatant, which were mixed thoroughly, and after 6 min, 2 mL 1 M NaOH was added, and 1 min later, the absorbance at OD_510_ was measured using an ultraviolet spectrophotometer (UV-6100, Metash Instruments Co., Ltd., Shanghai, China). All chemicals and reagents used in this experiment were of analytical grade and procured from Shanghai Hushi Chemical Reagent & Analysis Instrument Co., Ltd., Shanghai, China. Rutin (Shanghai Yuanye Bio-Technology Co., Ltd., Shanghai, China) was used as the standard to create a calibration curve ranging from 0.1 to 1.0 mg/mL for determining the flavonoid content. Each treatment comprised three biological replicates.

### 4.3. Determination of Total Lignin Content

Total lignin in tea leaves was extracted and measured as per a previously described optimization [49]. Initially, the tea leaves samples were pulverized into powder. Subsequently, 0.02 g of dried tea leaves was eluted with 1.75 mL deionized water, followed by the centrifugation of the mixture at 10,000× *g* for 5 min. Afterward, the supernatant was discarded, and the sediment was dissolved in 1.75 mL 99.8% methanol (Shanghai Hushi Chemical Reagent & Analysis Instrument Co., Ltd., Shanghai, China) at 60 °C in the water bath for 20 min, followed by cooling in ice and subsequent centrifugation of the mixture at 10,000× *g* for 5 min to obtain the sediment. The above procedure was repeated and eluted with methanol once and then the sediment was evaporated in a fume hood. The dry sediment was mixed with 1 mL 3 N HCl and 100 μL thioglycolic acid (Aladdin Scientific Corporation, Riverside, CA, USA). Later, the mixture was incubated at 80 °C for 3 h, cooled in ice, and centrifuged at 10,000× *g* for 5 min to obtain the precipitate. The precipitate was washed with 1 mL deionized water and centrifuged again, then dissolved in 1 mL 1 N NaOH, shaken for 16 h at 80× *g*. The supernatant was collected into a new tube after being centrifuged at 10,000× *g* for 5 min and 200 μL 12 N HCl was added. The mixture was cooled on ice for 4 h. After centrifugation at 10,000× *g* for 5 min, the sediment was dissolved again in 1 mL 1 N NaOH to complete the extraction. An amount of 1 N NaOH as control, 200 μL extraction was mixed with 1800 μL 1 N NaOH to measure the absorbance at OD_280_ using the ultraviolet spectrophotometer (UV-6100, Metash, Shanghai, China). The alkali lignin (Sigma-Aldrich, St. Louis, MO, USA) was used as the standard to make the calibration curve from 0 to 0.5 mg/mL. Each treatment had three biological replicates.

### 4.4. Quantitative Real-Time PCR Analysis

RNA samples were extracted from tea tree leaf tissues and reverse transcribed using the RNAprep Pure Plant Plus Kit (Tiangen, Beijing, China) following the manufacturer’s instructions. The primer sequences used are shown in Table S1. Quantitative real-time PCR (qRT-PCR) was performed using the SYBR Green Premix Pro Taq HS qPCR Kit (AG, Changsha City, China) on a Light Cycler 480II (Roche, Basel, Switzerland). The reaction system consisted of 20 μL, including 10 μL of SYBR Green, 7.2 μL of ddH_2_O, 0.4 μL each of forward and reverse primers, and 2 μL of DNA template. The qRT-PCR conditions consisted of a preliminary step at 95 °C for 1 min, followed by 40 cycles of denaturation at 95 °C for 10 s, annealing and extension step at 60 °C for 30 s. The glyceraldehyde-3-phosphate dehydrogenase (*GAPDH*) gene was used as an internal reference gene, and relative gene expression was calculated using the method of Livak et al. [50] to calculate mean 2^−ΔΔCT^ values.

### 4.5. Untargeted and Targeted Flavonoid Metabolomics Analysis in Tea Leaves

The collected tea leaves samples were lyophilized and ground into powder; 0.1 g was weighed and added with 75% methanol to grind well, processed by ultrasonic waves at 15 °C, centrifuged, and then the supernatant was filtered through a 0.22 mm filter membrane. Supernatants were analyzed using an UltiMateTM 3000 ultra-high-performance liquid chromatography system (Thermo Fisher Scientific Corporation, Lenexa, KS, USA). The chromatographic column used was Waters Acquity HSS T3 column (2.1 mm × 150 mm, 1.8 µm, Milford, MA, USA); the column temperature was set at 35 °C, with an injection volume of 3 µL and a flow rate of 300 µL/min. The mobile phases were 0.1% formic acid in water (A) and 0.1% formic acid acetonitrile solution (B). For the elution conditions and processing of raw data, we used the method of Zhu et al. [51]. Compounds were selected that were present in all QC samples with a relative standard deviation (RSD) of less than 25%. The relative abundance of metabolites was expressed as peak area and normalized. Compounds were categorized by comparison with the local and online databases (www.mzcloud.org (accessed on 20 March 2024); https://metaboanalyst.ca (accessed on 20 March 2024) and www.kegg.jp/ (accessed on 20 March 2024)) of standards and substances of concern were selected for analysis.

### 4.6. Data Analysis

Data were processed using Excel 2017 and statistically analyzed with SPSS 26.0 (Statistical Products and Services Solutions, IBM, North Castle, NY, USA) using Duncan’s Multiple Range Test at a significance level of *p* < 0.05. The data visualization was created by Origin 2021 (OriginLab Corporation, Northampton, MA, USA). All experiments were conducted with three independent biological replicates. The data were presented as the mean ± standard deviation SD of three biological replicates.

## 5. Conclusions

**Figure 5:**
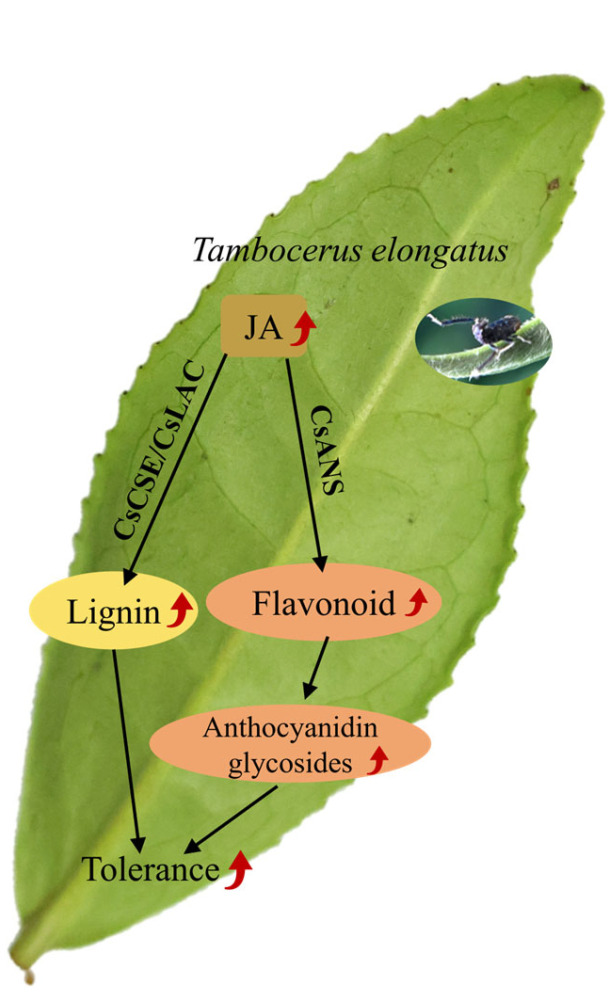
The sketch illustrates the response of *Camellia sinensis* L. to *Tambocerus elongatus* infestation through secondary metabolism and defense hormone pathways. When infested by *Tambocerus elongatus*, tea plants activate jasmonic acid, a hormone significantly associated with pest resistance. This activation triggers the induction of flavonoids and lignans, particularly lignin, in the secondary metabolic pathway, thereby enhancing the plant’s resilience against the pest. In this model, solid lines denote pathways that play a pivotal role, and arrows indicate up-regulation.

The response of tea plants to pest infestation is complex. *T*. *elongatus* is one of the pests that significantly affect the yield and quality of *Camellia sinensis*. This study elucidates the inherent resistance mechanisms of tea plants against *T. elongatus*, which manifest in the primary metabolism as a decrease in the amino acid content and an increase in the sugar content following infestation, aspects that have received less attention previously. The phenylpropanoid metabolic pathway, a crucial secondary metabolic pathway in plants, shows a significant increase in flavonoid and lignin content, with lignin showing a more pronounced increase. Among the flavonoid metabolites, there is a decrease in proanthocyanidins and an increase in anthocyanin glycosides (anthocyanins and their derivatives). Gene expression changes indicate the down-regulation of flavonoid biosynthesis genes and up-regulation of lignin pathway genes, suggesting a pivotal role for lignin in the tea plant’s response to pest damage. Additionally, JA signaling is primordially activated in response to *T. elongatus* damage and may positively regulate lignin synthesis. These findings suggest that lignin and JA are central to the tea plant’s defense against *T. elongatus*, with sugar production and the accumulation of anthocyanin glycosides being key components of the biotic stress response (Figure 5). In summary, this study provides a theoretical basis for understanding the defense mechanisms of tea plants against *T. elongatus* by elucidating the responses in both the primary and secondary metabolic pathways.

## Data Availability

The data presented in this study are available on request from the corresponding author.

## Supplementary Materials

The following supporting information can be downloaded at: https://www.mdpi.com/article/10.3390/plants14020260/s1, Table S1: Genes and primers used for qRT-PCR assays; Figure S1: Oxidative stress-induced damage in tea plants under biotic stress conditions. (**a**) MDA content of tea leaves in different infestation levels of *T. elongatus*. (**b**) H_2_O_2_ content of tea leaves in different infestation levels of *T. elongatus*.
