# Supplementary material for: Lignin Metabolism Is Crucial in the Plant Responses to Tambocerus elongatus (Shen) in Camellia sinensis L
Source: Plants (Basel). 2025 Jan 17;14(2):260. doi: 10.3390/plants14020260 (PMC11768230; doi:10.3390/plants14020260)
Supplement: Supplementary file 1 [file plants-14-00260-s001.zip › plants-3389621-supplementary.pdf]

## Supplemental matarial

**Table S1.** Genes and primer used for qRT-PCR assays

| Genes | Type | Primer sequence (5′-3′) |
|---|---|---|
| *GAPDH* | Forward | AGATCCCATGGGCTGAGACT |
| | Reverse | GAGACCCTCAACGATGCCAA |
| *PAL* | Forward | GAATGCCGGTCTTATCCACT |
| | Reverse | CGGTGAACACCTTGTCAAAC |
| *C4H* | Forward | CGAGAGGTTCTTGGAAGAGG |
| | Reverse | AGAATTGGCAGAGCAAGGAT |
| *4CL* | Forward | GGAGGTTATCCTGGACCTCA |
| | Reverse | GGCAAGCCTTGTAGTGTGAA |
| *CHS* | Forward | CTCAAACCATCCTCCCGGAC |
| | Reverse | GGGCTTAAGGGCCAACTTCA |
| *CHI* | Forward | CTGGTGGAAAGAATGACGAGC |
| | Reverse | TGTATCCTGGAAGCCGAGAAA |
| *F3H* | Forward | ATGCGGATCATCAGGCAGTA |
| | Reverse | CGAACGTGATTGGCTCTTCA |
| *F3′H* | Forward | GCCCAATGCTGATGTTAGGG |
| | Reverse | ATGGACCAAGGTCGCAGTTA |
| *F3′5′H* | Forward | CGACGGCTACAAGAATCCGA |
| | Reverse | ACATCAGGGTCTCGGCCTAT |
| *FNS* | Forward | CCCTTTGGTACTGGGAGGAG |
| | Reverse | ATCCCGGGTTGTTCAGTCAT |
| *FLS* | Forward | CAACATGTACCCACCATGCC |
| | Reverse | GAAGACCGGGAACGTCATTG |
| *DFR* | Forward | ATCAAGCCGACAATCAACGG |
| | Reverse | GGTTGTTGGTGTTCCTGGAC |
| *ANS* | Forward | CATTTGGCCCAAGACACCAA |
| | Reverse | GGAGGAGCTCTTCTTTGCCT |
| *ANR* | Forward | TTGCGCTGTCAATACCAGTG |
| | Reverse | ACGAGAGGATCAACTTCGCT |
| *LAR* | Forward | GCATCGTTCACACACGACAT |
| | Reverse | GCAGTGTTTCCATCCGTCTC |
| *UFGT* | Forward | CCGTAAGGAGGGTGATGGAC |
| | Reverse | GGACCCACCTTCTTTGACAGC |
| *HCT* | Forward | CCACACTTTCAAGTGCCCAA |
| | Reverse | CATTGGTTGGACTCGGCAAT |
| *C3H* | Forward | AAGCCACTGGTTGTCAGGAG |
| | Reverse | TGAACTCCTGCCCTTCTTCG |
| *C3′H* | Forward | GCTTCCAATCGTCGGCAA |
| | Reverse | CGAAACTATCACGTTCAGTGTCG |
| *CSE* | Forward | AGGTCCATCTGAAACAGCCA |
| | Reverse | GGTGGAACCATGTCATCTGC |

| | | |
|---|---|---|
| *COMT* | Forward | CTCACCATCTCCAACCTCGT |
| | Reverse | CATAGCGAACAAGCAAGCCT |
| *CCoAOMT* | Forward | GACGCTCCGATGAGGAAGTA |
| | Reverse | TTTGTCCAGCAATGGGTTGG |
| *F5H* | Forward | TAGACTCGCTAAGGCTCGTG |
| | Reverse | CATCATCGGCGCATGTACTC |
| *CCR* | Forward | TGCTCACCAGACTCACAGTT |
| | Reverse | GACGGCTGTCTGCAAATCAA |
| *CAD* | Forward | ATGGGCTGCAAGAGATCCAT |
| | Reverse | CAGCATAACACACTCCGCAA |
| *LAC6* | Forward | ACCCAATTCATCTCCACGGT |
| | Reverse | ACAGTGCATGAACCAAACCC |
| *PER* | Forward | GAGAAAGGGTCTGCTCGTGC |
| | Reverse | CATTTGGGATGTGCTCTTTGAC |
| *CsLOX2* | Forward | GGCAGTGAAATTCAAGGTTAGAG |
| | Reverse | TTCTTTGGGGCTTTCGTTT |
| *CsLOXC* | Forward | TGAGCAGGGTGTTGAAGGC |
| | Reverse | CATTGTATCACGGAAGTGAGGAT |
| *CsAOC* | Forward | CTGCCTCCTCTGCTACACTCA |
| | Reverse | TCTTGAAGCGTCAGATGGGT |
| *CsOPR3* | Forward | CGATCAACAGCCGGTGGATTT |
| | Reverse | GCGTGGACAGCATCAACCAC |
| *CsJAZ1* | Forward | TAAAACCGCCCCAATGACGA |
| | Reverse | TTCCGAGGGCTGGAATAGGA |
| *CsMYC2a* | Forward | CGCAGATATTCAACCAGGAGAG |
| | Reverse | ACGGAGGATTGCCAGAAGA |
| *CsMYC2c* | Forward | CACCACCAACACTCCAACAA |
| | Reverse | GCCGTCAAGCCAAGAGAG |
| *CsNPR1* | Forward | CATCGCTCAGAAATGGTGT |
| | Reverse | CACGTCGAGCGGAGTAATTC |
| *CsICS1* | Forward | AACGAGTACATGCCCAATAAGAC |
| | Reverse | TCAGCCTGTAAACAAGCCAAC |

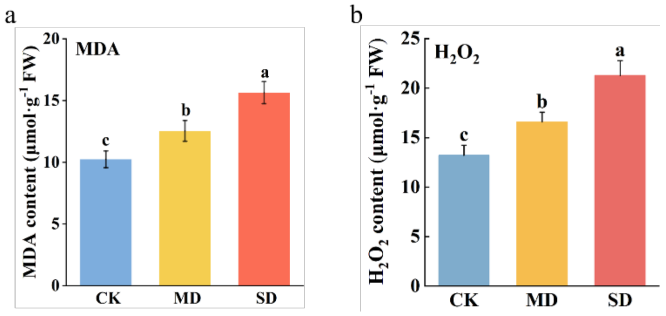


**Figure S1:** Oxidative stress-induced damage in tea plants under biotic stress conditions. a. MDA content of tea leaves in different infestation levels of *T. elongatus*. b. $H_2O_2$ content of tea leaves in different infestation levels of *T. elongatus*
